# Fosfomycin: a good alternative drug for prostate biopsy prophylaxis the results of a prospective, randomized trial with respect to risk factors

**DOI:** 10.1590/S1677-5538.IBJU.2016.0619

**Published:** 2017

**Authors:** Erdem Kisa, Mustafa Ugur Altug, Oguz Alp Gurbuz, Harun Ozdemir

**Affiliations:** 1 Department of Urology, Tepecik Education and Research Hospital, Izmir, Turkey;; 2 Department of Urology, Acibadem University School of Medicine, Istanbul, Turkey;; 3 Department of Microbiology, Diskapi Yildirim Beyazit Education and Research Hospital, Ankara, Turkey;; 4 Department of Urology, Haseki Education and Research Hospital, Istanbul, Turkey

**Keywords:** Fosfomycin, Prostate, Biopsy

## Abstract

**Purpose:**

To determine the risk factors and the efficiency of rectal swab samples to prevent infectious complications in prostate biopsy, and compare fosfomycin with ciprofloxacin use in prophylaxis.

**Materials and Methods:**

Between May and October 2014, pre-biopsy risk factors and their effect in ciprofloxacin and fosfomycin prophylaxis were determined. Pre-biopsy urinalysis, urine culture and rectal swab samples were obtained from all of the patients. Rectal swabs were obtained upon admission, and biopsy was performed in the following 3-7 days. The place of rectal swab samples and efficiency of fosfomycin use was evaluated.

**Results:**

Pre-biopsy rectal swabs were obtained from 110 patients who revealed 60.9% fluoroquinolone resistance (FQR), and 32.7% fluoroquinolone sensitivity (FQS). Fosfomycin resistance was present in 3 patients. Ciprofloxacin use in last 6 months was the only risk factor for FQR. Antibiotic prophylaxis was given to both groups with and without risk factors, according to swab results, and no infective complications were observed. Among the group where fosfomycin was used empirically, one patient had an infection needing hospitalization, however this constitutes no statistical difference between the Group that fosfomycin used empirically or according to swab results (p=0.164).

**Conclusions:**

In prostate biopsy prophylaxis, ciprofloxacin may be used liberally in patients without risk factors, but it should be given according to the rectal swab results in the patients with risk, and fosfomycin may be used independently of risk factors and rectal swab results.

## INTRODUCTION

Prostate cancer (PCa) is an important disease because of its high incidence and being the second cancer mortality cause (1). Transrectal ultrasound guided biopsy (TRUSG-Bx) is a generally accepted standard method for diagnosis of PCa. Infection is the most serious complication of biopsy. It is mostly afebrile, non-complicated (1.2-11.3%), but rarely it can become pyretic (1.4-4.5%), may cause severe sepsis (0.3-3%), needing hospitalization and lead to a life threatening condition (2). Ciprofloxacin is widely used in TRUSG-Bx prophylaxis. Up to 20% increase in the fluoroquinolone resistance (FQR) in rectal swab samples and the observation of the FQR bacteria in about 50% of the infections have created a need for alternative prophylaxis (3). For TRUSG-Bx prophylaxis, fosfomycin may be preferred, because it's more reliable than the fluoroquinolones. It has lower resistance rate and oral single-dose usage (4).

The aim of this study is to consider FQR in order to determine the risk factors prior to TRUSG-Bx as well as to determine the reliability of taking rectal swab samples, and to compare the efficiency of fosfomycin and ciprofloxacin prophylaxis.

## MATERIALS AND METHODS

Between May and October 2014, 110 patients were included in this study, for which TRUSG-Bx was planned because of PCa suspicion. The patients were informed about the study, and written consents as well as local ethics committee decision were obtained. Pre-biopsy urinalysis, culture and rectal swab samples were obtained from all patients. For the antibiotic prophylaxis, the patients were divided into 2 main groups according to risk factors (ciprofloxacin or other antibiotic use in the last 6 months, diabetes mellitus (DM), urethral catheterization, genitourinary system (GUS) operation history).

Group A included patients with no risk factors. It was divided into 2 sub-groups: patients using single dose fosfomycin the night before the biopsy (A1), and those using ciprofloxacin twice daily for 5 days, beginning the day before (A2). In both groups the prophylaxis was started before getting the swab results.

**Figure 1:**
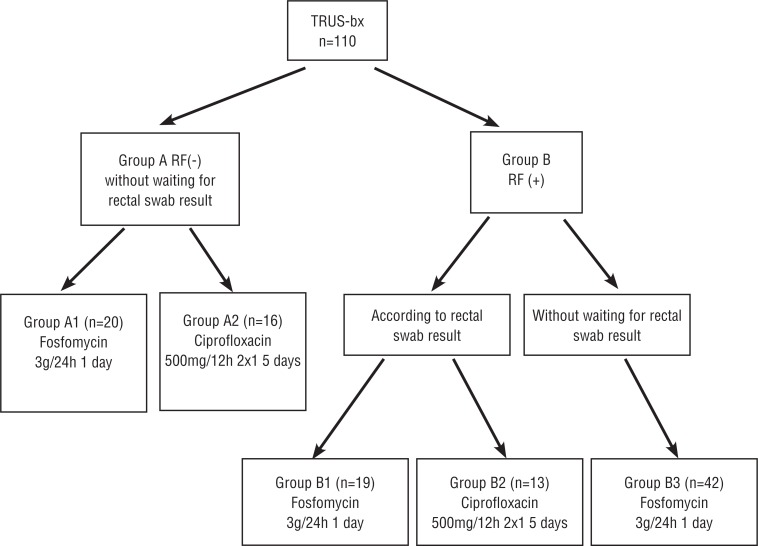
Study Design (Algorithm of TRUSG-bx patients). **RF =** risk factors; **TRUSG-BX =** transrectal ultrasound guided biopsy; **UTI =** urinary tract infection

Group B included those with risk factors. It was divided into 3 sub-groups: patients who took fosfomycin (B1) or oral ciprofloxacin (B2) according to the swab results, and those who took fosfomycin (B3) empirically (Figure-1).

Rectal swabs were obtained upon admission, and biopsy (standard 12 quadrant) was performed in the following 3-7 days. Using Kirby-Bauer disc diffusion method, in line with the suggestions of the “Clinical and Laboratory Standards Institute (CLSI)” fosfomycin and ciprofloxacin sensitivities of Escherichia coli (E. coli) were examined.

The patients were contacted by telephone 24 hours after biopsy, and were asked if they had fever, and their conditions were evaluated by urinalysis and cultures in the 1^st^ and 4^th^ weeks.

### Statistical Analysis

Data was analyzed using SPSS for Windows. Risk factors predicting the FQR were determined using multivariate logistic regression analysis. Odds ratio and 95% confidence intervals for each risk factor were calculated. The results with p <0.05 were considered significant.

## RESULTS

Between May and October 2014, pre-biopsy swabs were obtained from 155 patients. Following the evaluations of the patients, 110 were included in the study and 45 excluded because of contamination.

Mean age of the patients was 63.8. Mean PSA was 13.3ng/mL 18 patients had repeated biopsy, and 7 had indwelling catheter. Among the comorbidities 16 had diabetes mellitus. When the swabs were evaluated E.coli had grown in 93.6% (103/110). FQS was present in 67 (60.9%), and FQR in 36 (32.7%). Fosfomycin resistance was seen in 3 (2.7%), sensitivity in 100 (90.9%). When the risk factors were evaluated, they were negative in 36 (32.72%), and positive in 74 (67.27%) patients.

FQR was present in16.7% in Group A (6/36), and in 40.5% in Group B (30/74). The increase in FQR of Group with positive risk factors was found to be statistically significant (p=0.012). 30 of 36 patients with FQR and 39 of the 67 patients with FQS had risk factors. The risk factor positivity was significantly associated with the FQR (p=0.010). Ciprofloxacin use in the last 6 months was the only risk factor for FQR (p=0.002); 17 of the 36 patients (47.2%) who had FQR, and 12 of the 67 patients (17.9%) who had FQS used ciprofloxacin in the last 6 months, which increased FQR 4.10 times (95% CI: 1.66-10.13). When the relation of diabetes mellitus and FQR was evaluated, DM was present in 25% of the patients with FQR, and in 10.4% with FQS. Although the presence of DM was not statistically significant it increased the FQR risk by 2.86 folds (95% CI: 0.96-8.47) (p=0.052). No association was observed between the antibiotic use (except ciprofloxacin) and catheter history (p=0.394 and p=0.142). In the analysis of the 6 patients who had GUS operation history FQR was not detected in the rectal swab.

In the multivariant analysis of the risk factors the most determining factor for the FQR in swab was the use of ciprofloxacin in the last 6 months and was an independent risk factor.

There was no statistically significant difference, in terms of UTI, between A1 and A2 Groups without risk factors (p=1.000) and fosfomycin or ciprofloxacin can be used safely in these patients.

There was no difference, in terms of UTI, between the fosfomycin (A1) Group which had no risk factor and received prophylaxis without the swab result and the fosfomycin (B1) Group with risk factors and received prophylactically according to the swab result (p=0.487). Also, there was no statistically significant difference between the groups empirically using fosfomycin (Group B3 and A1) with and without risk factors (p=1.000). Whether the presence of risk factors in the patient has an effect on the fosfomycin use could not be shown. However, the observed 4 infections without fever in Group B3 were explained as increased asymptomatic bacteriuria risk due to in-dwelling catheter (p=0.002).

There was no difference, in terms of UTI, between the fosfomycin Group B1 with risk factors and received prophylaxis according to the swab, and Group B3 with risk factors using fosfomycin empirically (p=0.164). It was concluded that checking the rectal swab before fosfomycin prophylaxis was not necessary in terms of decreasing UTI.

There was no difference, in terms of UTI, between the ciprofloxacin Group A2 without risk factors and received prophylaxis without checking the swab, and the B2 group with risk factor and received prophylaxis according to ciprofloxacin sensitivity in the swab (p=1.000). It was understood that the patients who developed UTI in both Groups had asymptomatic bacteriuria due to indwelling catheter.

When we examined the 30 patients in the Groups B1, B2, and B3, all with risk factors, we found that 56.7% (n=17) with FQR and 34.3% (n=23) with FQS used ciprofloxacin in the last 6 months and the effect of this on the FQR was not different between the Groups (p=0.235). However, it was understood in the multivariate analyses that the ciprofloxacin exposure affected FQR [OR=2.839, 95% CI: 1.055-7.640, p=0.039]. On the other hand, DM, catheter history, antibiotic use, GUS operation history did not affect FQR in these Groups.

**Figure 2:**
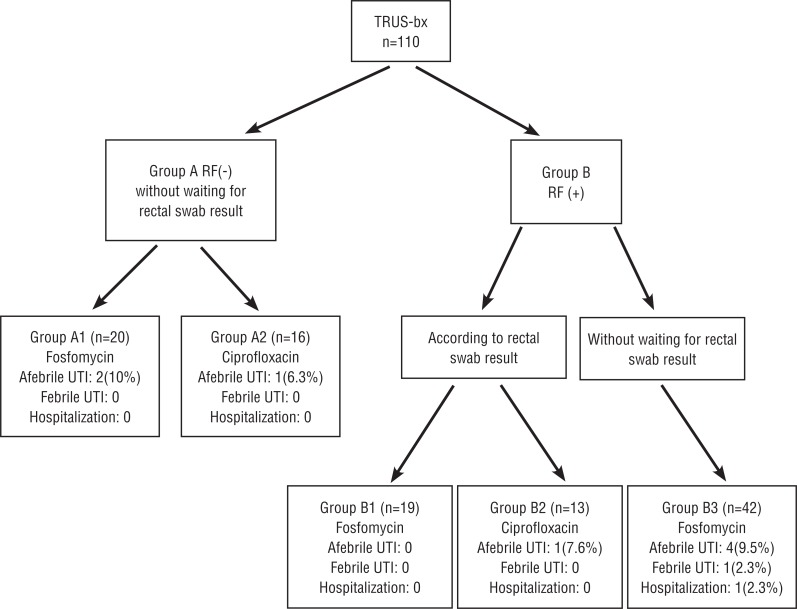
Infectious complication rates of the patients in the study Group. **RF =** risk factors; **TRUSG-Bx =** transrectal ultrasound guided biopsy; **UTI =** urinary tract infection

Infectious complications occurred in 10 (9%) patients. There was asymptomatic bacteriuria in 5 (4.5%), UTI without fever in 3 (2.7%), and fever in 2 patients (1.8%) (Figure-2). The urine cultures of the patients with asymptomatic bacteriuria was repeated after 2 weeks and treatment was initiated in the ones with bacterial growth. UTIs without fever were treated according to urine cultures.

## DISCUSSION

PCa is the most prevalent solid tumor in Europe and diagnosed mainly by TRUSG-Bx. There is not yet a standard antibiotic prophylaxis protocol, but mostly fluoroquinolones are used (1). In a study, it was revealed that fluoroquinolones were used in 2 million prostate biopsies every year (5). In another study, antibiotic sensitivity of E. coli was evaluated. It was shown that ciprofloxacin resistance rate of 0.8% in 810 E. Coli strains between 1994 and1996, has climbed to 12% in 1163 E. Coli strains between 2000 and 2002 (6).

The increase in the infectious complications despite prophylaxis has been associated with the presence of ESBL positive bacteria and especially to the FQR in the rectal swab (7). While in the initial studies of FQR in rectal swab in 2010 it was found that FQR was only 10.6%, in the more recent studies it was shown to increased up to 22% (8–9). In our study, FQR rate in swab was found in 32.7% (36/110), which is higher than other studies (10–12). Because the most determining risk factor for the post TRUSG-Bx infectious complications is FQR bacteria and because the FQR rate in our region is high, it seems necessary to review the ciprofloxacin use in prophylaxis (11).

In the largest prospective study on FQR, swab of 849 patients were examined and the resistance rate was found 19% (n=161), with the most determining factor for FQR being fluoroquinolone use in the last 3 months and the patients with heart valve prosthesis. FQR patients comprised 48% (15/31) of all infectious patients. With this result, it was emphasized that it may be beneficial to determine FQR in rectal swab and to start targeted antibiotic prophylaxis (TAP) or TAP could be used by considering the antibiotic profiles in the swabs (12).

In our study, having a GUS operation or repeat biopsy didn't affect FQR, although they used ciprofloxacin during these procedures. The reason for this might be the time that passed after the ciprofloxacin exposure.

It was shown in many studies that FQR bacteria present in swab increases the infection and sepsis rates. Liss et al. in their meta-analysis, have determined the FQR rate was 20.5% (n=549) in 2673 patients. The difference of infection rates of FQR and FQS were found to be 6.6% and 1.1%, and hospitalization rates were 4.4% and 0.9% respectively. Both results were statistically significant. Among the patients who received fluoroquinolone prophylaxis the infection rate was 8.2% in those with FQR and 1.8% in those with FQS. In the same study, it was shown that the presence of FQR organisms in the swab increased the infection and hospital admission rate 3 times, and the presence of FQR organisms in both swab sample and fluoroquinolone prophylaxis increased the hospital admission 6 times. The presence of FQR bacteria in swab was the risk factor contributed the most to the infection rate increase. In FQR (-) patients fluoroquinolone prophylaxis would be sufficient and the infection rate remains in 1% (3).

When the FQR and FQS patients in our study were evaluated, there were 5 UTIs with 2 asymptomatic bacteriuria among the 67 patients in FQS, and 4 UTIs with 2 asymptomatic bacteriuria among the 36 patients in FQR Group. There was no statistically significant difference between the Groups (p=1). It was shown in many studies that FQR being an independent risk factor in terms of UTI in the patients receiving ciprofloxacin prophylaxis (3–10). However, in our study, out of 6 patients with FQR in Group A, 5 of them received fosfomycin and only 1 patient received ciprofloxacin prophylaxis. Furthermore, the patients with FQR in Group B1 and those with FQR in B3 who took fosfomycin prophylaxis, lead to low infection rates like that in FQS patients. Thus, we conclude that infectious complication rates could be decreased by not giving ciprofloxacin to the patients who have or with risk of having FQR in swab.

To decrease infectious complications, the use of antibiotics according to the rectal swab is a promising method. In the study by Taylor et al. Fluoroquinolone was started according to fluoroquinolone sensitivity in the swab in 112 patients, and empirically in 345 patients without taking swab. Among the 112 patients 19.6% had FQR in swab. Ciprofloxacin was used in the patients with FQS and various prophylaxis (TMP/SMX, cephalosporin) in those with FQR. While no infectious complications were observed in any of these 112 patients, 9 (2.6%) infectious complication and 1 sepsis were observed in the other group. This study was exciting and promising with the TAP in TRUSG-Bx prophylaxis. In the same study, it was suggested that using fluoroquinolone in prophylaxis of patients with FQR risk was no longer logical. Another point mentioned was that the risk of complication due to FQR increased in 68 of the 345 patients (19.6%), and infectious complication was not observed only in 9 patients (13%). It was emphasized that the infections following TRUSG-Bx were not only due to FQR in the swab or the preference of the antibiotic, but also to some other factors (humoral immunity, procedure technique, bacterial inoculums). Taylor et al. stated in their study that it was necessary to obtain 38 rectal swabs in order to prevent 1 infectious complication, but despite this, TAP was beneficial in terms of cost (10).

Liss et al. in their meta-analysis, stated that infectious complication risk would increase when the patients with risk of FQR would take fluoroquinolone prophylaxis, and starting TAP according to swab would be beneficial for those patient. Furthermore, in the future it wouldn't be possible for everybody to take the same antibiotic for prophylaxis (3). In our study, we used TAP to the 12 patients in Group B2 with risk factor and FQS according to swab, and did not observe any infection except one patient with indwelling catheter, who showed asymptomatic bacteriuria.

Fosfomycin, was first used by Ongün et al. they compared single dose fosfomycin with ciprofloxacin 2x500mg and levofloxacine 500mg. They found that it is an alternative prophylaxis and is effective against FQR bacteria, moreover it is easily used and well tolerated (4). In another study, Lista et al. compared fosfomycin and ciprofloxacin for prophylaxis. No significant difference was observed in terms of infection and sepsis, and it was emphasized that fosfomycin may decrease FQR related infections (13).

In our study, there was no significant difference, in terms of infection, between the A1 and the B1 fosfomycin groups, which reveals that fosfomycin prophylaxis could be used independently of risk factors. Moreover, this may also prove that fosfomycin could be preferred to ciprofloxacin in patients who had FQR. The observation of asymptomatic bacteriuria in the 4 patients with indwelling catheter in the B3 Group can be explained with fosfomycin not staying enough in the bladder to show its effect (it reaches maximum concentration in 4 hours).

## CONCLUSIONS

In this study, it was shown that using fosfomycin empirically or according to the rectal swab, regardless of patients having or not having risk factors, does not affect infection rates. There is no need for rectal swab sampling if fosfomycin prophylaxis is planned. The result was the same for ciprofloxacin Group who had no risk factor; we found similar infection rates which shows that both prophylaxis could be used in the patients without risk factors. But if ciprofloxacin prophylaxis is planned for the patients with risk factors, it must be used according to rectal swab results and infection rates may decrease with targeted antibiotic prophylaxis.

When fosfomycin and ciprofloxacin were compared in terms of cost effectiveness, considering increase in the infection rates due to ciprofloxacin, it could be suggested that fosfomycin would become more economical in the long run.

## Abbreviations

FQR: = Fluoroquinolone-resistance
FQS: = Fluoroquinolone-sensitivity
DM: = Diabetes mellitus
UTI: = Urinary tract infection
GUS: = Genitourinary system
E.coli: = Escherichia coli
PCa: = Prostate cancer
TRUSG-Bx: = Transrectal ultrasound guided biopsy
TAP: = Targeted antibiotic therapy
